# Phenotypic determinism and contingency in the evolution of hypothetical tree-like organisms

**DOI:** 10.1371/journal.pone.0211671

**Published:** 2019-10-31

**Authors:** Tomonobu Nonoyama, Satoshi Chiba

**Affiliations:** 1 Graduate School of Life Sciences, Tohoku University, Katahira, Aoba-ku, Sendai, Japan; 2 Center for Northeast Asian Studies, Tohoku University, Kawauchi, Aoba-ku, Sendai, Japan; Universitetet i Bergen, NORWAY

## Abstract

Whether evolutionary history is mostly contingent or deterministic has been given much focus in the field of evolutionary biology. Studies addressing this issue have been conducted theoretically, based on models, and experimentally, based on microcosms. It has been argued that the shape of the adaptive landscape and mutation rate are major determinants of replicated phenotypic evolution. In the present study, to incorporate the effects of phenotypic plasticity, we constructed a model using tree-like organisms. In this model, the basic rules used to develop trees are genetically determined, but tree shape (described by the number and aspect ratio of the branches) is determined by both genetic components and plasticity. The results of the simulation show that the tree shapes become more deterministic under higher mutation rates. However, the tree shape became most contingent and diverse at the lower mutation rate. In this situation, the variances of the genetically determinant characters were low, but the variance of the tree shape is rather high, suggesting that phenotypic plasticity results in this contingency and diversity of tree shape. The present findings suggest that plasticity cannot be ignored as a factor that increases contingency and diversity of evolutionary outcomes.

## Introduction

There has been much debate as to whether the evolutionary history of life is mostly contingent or more or less deterministic [1–4]. A metaphor of “replaying life’s tape” [1] was used to emphasize the preeminent role of contingency in the evolutionary process [1]. In this view, the outcome of evolution could be dramatically different if it was replayed, because evolution is essentially a stochastic phenomenon whereby trajectories that start close to each other soon diverge. Experimental study of bacteria has suggested that different experiments might lead populations along different evolutionary paths [5]. Empirical and theoretical studies suggest that natural selection constrains organisms to a relatively few highly adaptive options [3]. Furthermore, constraints might come from developmental, processes and environmental, physical and chemical biases[6]. In these views, the evolutionary routes are many, but the destinations are limited. In the present study, we examine relative contributions of determinism and stochasticity in evolution. This has not been addressed directly, and the quantification of the predictability of evolution remains elusive.

The concept of a fitness landscape [7] has been used to address this issue. This concept has influenced many research fields on evolution and much effort has been spent to understand the characteristics of empirical landscapes.[8–10]. The base concept of a fitness landscape assumes that there is a functional relationship between the genome of an organism and its growth rate and fitness [11]. The model of the fitness landscape describes how some phenotypes are more likely to evolve than others, and how developmental mechanisms could limit the evolutionary change [12]. In this situation, multiple evolutionary trajectories are accessible, but evolution might be strongly constrained to a particular adaptive peak [13].

However, in many real-world scenarios, fitness evaluations are not trivial [14] and experimental evolution involving sexual reproduction and multicellular organisms with long life timescale is difficult [15], so that sometimes it is difficult to apply the fitness landscape into real biological systems. Also, phenotypic changes are often largely affected by phenotypic plasticity. The shape of the adaptive landscape might largely be affected by phenotypic plasticity, while such effects of plasticity have not been considered in the studies of repeatability of phenotypic evolution. In the present study, we simulate parallel evolution experiments that focus on the predictability of evolution while also considering the effects of plasticity.

To address this subject, a community of dendritic organisms (e.g., tree, coral, sponge, Stromatoporoidea) provides an excellent model [16–19]. We assume hypothetical tree-like organisms and simulate their evolution and plastic change using the Honda model [20]. Using this model, we construct an individual-based model in which individuals compete with each other for light. We assume organisms are growing under direct ambient light, and we are not strictly concerned about the weight of their body. The morphology of the organisms was assumed to be determined by plastic change as a response to environmental condition and by genetic change as a result of genetic drift, mutation, and natural selection. For example, the morphology of coral is determined by both environmental factors, particularly light environment [21,22] and genetic components [23]. We assume that the common evolutionary patterns can be found by simulating the shape of the hypothetical tree-like organisms [24].

This study aims to explore the factors that affect the evolutionary contingency and determinism. We test whether, after sufficient time, the same phenotypes evolve in the model community within every simulation. Particularly, we focus on how mutation rates affect the contingency of the evolutionary history and how plasticity is associated with the phenotypic diversity patterns.

## Methods

Here we constructed an individual-based model, in which dendritic-shaped individuals compete for light with other individuals and evolve through mutation and natural selection. Each individual was described by simple branching rules and parameters (e.g., Honda 1971). Using these models, the optimal branching rule to minimize the overlapping of leaf clusters was obtained [25,26]. For convenience, the organism in the model was called a “tree” and its single component was called a “branch”. In the present tree model, we tentatively call the clusters of trees a “forest”.

In this model, the branch development process was deterministic. The variety of tree shapes was produced by the growth of branches to adapt to the surrounding light environments. To describe how leaves receive light and how it inhibits other leaves from receiving light, a leaf ball model was used [27]. This model considers a leaf cluster as a ball and the branch according to the brightest vector. The influence of the local light environment on branch growth is an essential process determining the dynamics of tree crown architecture [28,29]. Using these methods, we developed the simulation model, incorporating the dependence of the number and size of new shoots on the photosynthetic production of parental shoots. This model describes the growth of trees by branch developments, which is determined by the light condition. Accordingly, in this study, we used a previous 3D tree growth model [20,30] by incorporating a leaf ball model and reaction process of branch developments against the photosynthetic condition.

### Tree growth

The tree morphology is determined by 11 parameters (Table 1). These parameters are all used by Honda[20] and Yoshizawa [31] and the basic relationships between these parameters and the tree shape were shown in these papers. Thus, details of this relationship were not analyzed in this paper. The exact description of the branching geometry is as follows. A parent branch diverges into two offspring branches through one branching process. The bifurcation process is repeated 5 times until the growth is over. The length of each of the two offspring branches is determined by the ratio of *R*_1_ and *R*_2_, respectively, to the length of the parent branch. The angle between the mother branch and two offspring branches are described as *θ*_1_ and *θ*_2_ respectively. In the case of the trunk, when the *θ*_1_ or *θ*_2_ equal to zero, one of the offspring branches grows vertically. Another branch was assumed to form a divergence angle alpha with the sister branch of its parent branch (i.e., the offspring branch is rotated with angle alpha around the trunk from the sister branch of its parent branch).

**Table 1:** Parameters used in the simulation.

Parameter	Description	Range
θ_1_	Branching angle 1	0≤θ_1_≤*π*
θ_2_	Branching angle 2	0≤θ_2_≤*π*
br_1_	Branch attenuation rate 1	0<br_1_
br_2_	Branch attenuation rate 2	0<br_2_
α	Rotation angle	0≤*α*≤*π*
*lr*	Leaf radius	0.0<*lr*
*la*	Leaf absorption rate	*la*<1.0
*cr*	Tendency to move	−1.0<cr<1.0
*at*	Avoid threshold	0.0<*at*<1.0
*ra*	Rate of avoid reaction	0.0≤*ra*≤1.0
*dt*	Death threshold	0.0<*dt*<1.0

It is assumed that the branches search for the direction of light in the surrounding environment. To describe a cluster of leaves, this model places a ball at the distal end of each branch. This ball is used to determine the brightest direction by making a map of the shadow of the other branches on the ball surface. The direction in which the branches will grow was determined according to the incoming light vector. ***V*** is the average vector of light. ***V*** is calculated by the following equation (Kanemaru et al, 1992):

$$V=(\sum_{n}\sum_{m}l(n,m)N(n,m)S(n,m))/|\sum_{n}\sum_{m}l(n,m)N(n,m)S(n,m)|$$

where *l*(*n*,*m*) is the brightness per area, ***N***(*n*,*m*) is the vector from the center of the ball to each cell on the ball surface, and *S*(*n*,*m*) is the area on the ball surface. n and m describe longitude and latitude. The light amount (*L*_*e*_) received by the leaf is calculated by the following equation: *L*_*e*_ = ∑_*n*_∑_*m*_*l*(*n*,*m*)*S*(*n*,*m*). Once the amount of the light received by the tree is calculated, branches adjusted their positions according to *V*. The considered branch is rotated around the point where it attaches to its parent branch, toward brightest direction. The actual angle used for changing branch direction, *ϕ*, is calculated by *ϕ* = *crδ*, where *δ* is the angle between ***V*** and original direction of the branch to grow. *cr* is the constant rate of bending the branch. In the case of *cr* = 0.0, there is no influence of the phototropism in the simulation. After all branches change their direction, those branches that cannot capture sufficient light do not extend and become lost. Even if the branches can capture sufficient light to grow, the growth rate of the sister branch changes with the ratio of *ra* [31]. The amount of light captured by an individual tree is calculated by summing all light captured by the survived branches of the individual tree. The light amount captured by the *Nth* tree is calculated by the equation *L*_*N*_ = ∑*L*_*e*_. Trees are placed in a square area at an even interval. The ground of the area is assumed to reflect light at a fixed rate. Light is considered as ambient light.

### Reproduction and evolutionary process

The simulation area is divided into the number of square cells. Each tree grows within a cell of the simulation area. The trees produce seeds and have the same lifespan. There is no overlap of generations among individuals. In the model, we assume that one tree produces one seed (offspring), and individuals with low fitness cannot produce seed (offspring). The total number of individuals is assumed to be constant.

We assume that the trees are all clones, and the 11 variables determining tree morphology are the same between offspring and its mother, except in cases of mutation, which occurs with a defined probability. A change of the value of each variable by mutation is described by adding a random value having a Poisson distribution with a mean of 0 and a standard deviation of 1.

After each generation, all of the cells are assumed to become empty. All offspring are ranked by fitness. Offspring in the bottom 20% rank do not survive. Each cell is placed by an individual randomly chosen from the survived offspring. If the fitness is equal, the offspring is selected at random and placed on the cell. The fitness *F* of the *Nth* individual is calculated using the following equation: *F*_*N*_ = *L*_*N*_*X*_*N*_(K−*X*_*N*_)*A*_*N*_(P−*A*_*N*_) where K and P are the constant, *L*_*N*_ is the light amount captured by *Nth* individual, *X*_*N*_ is the whole length of the individual, and *A*_*N*_ is the value calculated by multiplication of the size of the tree and efficiency of the light. Durable construction and efficiency cannot be achieved at the same time [32]. In this paper, we consider the durability of leaves to be correlated with the size of leaves; because it seems that a large leave needs a structure to support it.

### Phenotypic difference

We evaluated the tree phenotypes using two parameters, the aspect ratio of the branches and the number of branches of each individual. These two parameters are considered to be independent values. These parameters were calculated for all individuals, and their variance and average were obtained. Also, we examined changes in 11 genetically-determined variables, and calculated the average and variance for each variable. After sufficient generation times, the phenotypes of the evolved trees were compared among the different simulation runs, and thus, we can investigate if similar phenotypes evolve independently in the different simulation runs. At the end of each simulation run, the values of the above two parameters were plotted in the two-dimensional space.

We compare the patterns and position of the plots among different simulation runs. We calculated overall phenotypic difference *D* among different simulation runs with same values of the parameters, and used *D* as an indication of repeatability of the simulation run. Thus, low *D* implies that the simulation tended to yield the same result. The overall phenotypic difference between the *Ath* simulation run and the *Bth* simulation run are calculated as $D_{AB}=\sum_{n}^{N\epsilon A}\overset{⃑}{{|d}_{n}}|/N$, where ***d***_***n***_ is the shortest vector from *nth* plot in the *Ath* run to the *mth* plot in the *Bth* run. The average of the shortest distance of this vector represents the overall phenotypic difference between the different simulation runs.

## Results

**Fig 1:**
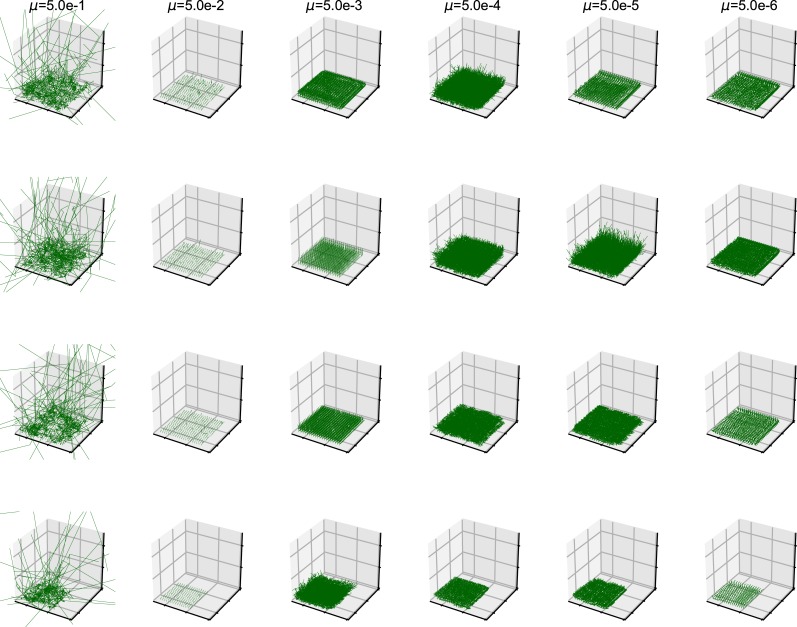
The 3-D shape of the results. Each map is one experiment result. The six columns represent the mutation rates. The mutation rates were 5.0e-1, 5.0e-2, 5.0e-3, 5.0e-4, 5.0e-5, 5.0e-6, from the left. The three rows represents the individual number. The bottom row is the result which contains 225 individual and other rows are in the result containing 400 individuals. All results are after 2500 time steps.

**Fig 2:**
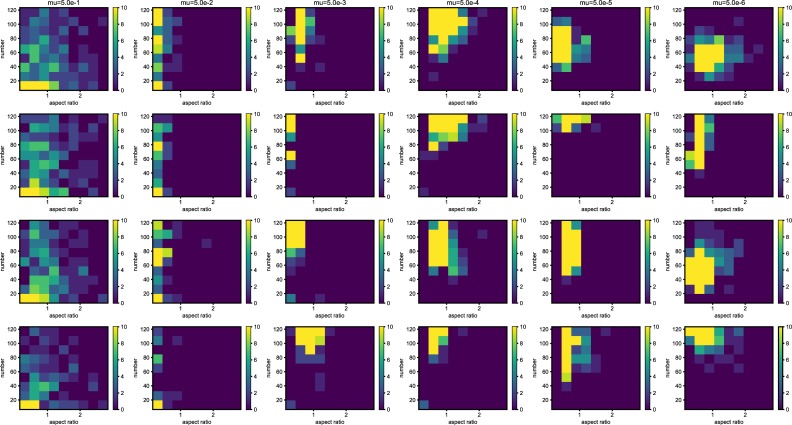
The histogram of the whole result. Each map is one experiment result, and the horizontal axis represents aspect ratio, which is calculated by *D*_*v*_/*D*_*h*_, where *D* is the distance from the root to the most furthest branch, and small v and h denote the horizontal distance and vertical distance. The vertical axis represents the number of the branches in the individual. The six columns represent the mutation rates. The mutation rates were 5.0*e*−1, 5.0*e*−2,5.0*e*−3, 5.0e−4,5.0e−5,5.0e−6, from the left. The three rows represents the individual number. The bottom column is the result which contains 225 individual and other rows are in the result containing 400 individuals. All results are after 2500 time steps. The color bar of the figures are limit at 10 individuals.

**Fig 3:**
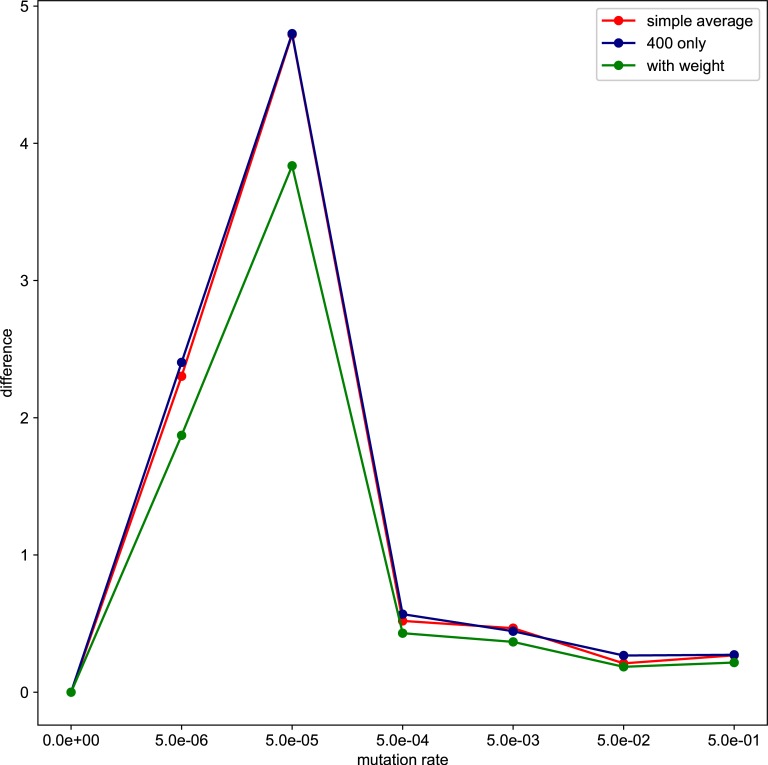
The average of the minimum distance. The horizontal axis represents the mutation rate. The vertical axis represents the average of the minimum distance cases of the same mutation rate. The green line represents for the simple average of four cases including results in 400 individuals and 225 individuals, and the blue line represents for the average which is considering the number of the individuals and weight. The orange line represents for the result only in 400 individuals. If the simulations all end at the same result, all points in the histogram become the same, and the minimum distance equals zero because all points are overlapped.

The shape of the organisms after 2500 generation is shown in Fig 1. We examined the aspect ratio and number of the branches of the all trees generated after 2500 generations (Fig 2). The number of types with different values for the aspect ratio of the branches and number of branches was larger with the higher mutation rate, and every individual has unique values for these traits at *μ* = 5.0*e*−1. Due to plasticity, there is no fixed form tree. The shape of the individuals is highly influenced by their surrounding environment. The relationship between mutation rate and average minimum distance of the phenotypic distribution among the different simulation runs (*D*) is shown in Fig 3. The differences in the phenotypic distribution among the different simulations (*D*) represented a hump-shaped relationship with the mutation rate, but its peak is located near the lowest mutation rate (5 × 10^−5^). The difference became the lowest at the highest mutation rate.

**Fig 4:**
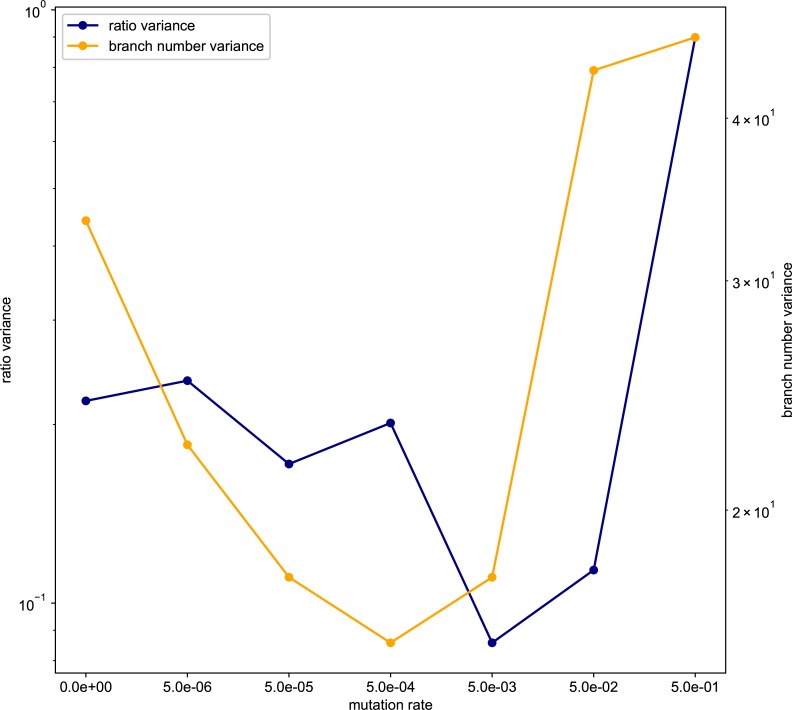
The Variance of the result in aspect ratio and branch number. The horizontal axis represents the mutation rate. The left vertical axis represents the aspect ratio dispersion, and the right vertical axis represents the branch number dispersion. Both lines are a downward convex.

**Fig 5:**
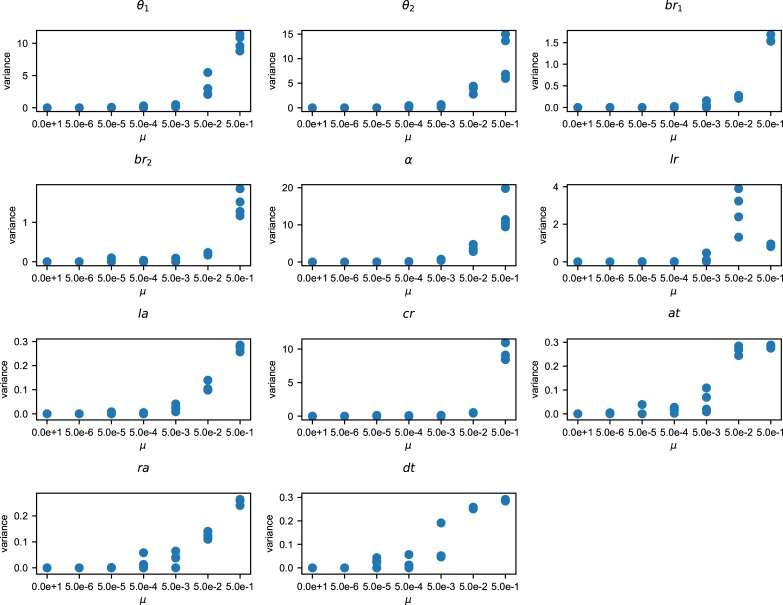
The variance of the parameters. The parameter meanings are shown in Table 1. The horizontal axis represents the mutation rate. The vertical axis represents the variance of each parameter.

Both of the variances of the aspect ratio of the branches and number of the branches became the lowest at the intermediate level of mutation rate (Fig 4). In contrast, the variance of the 11 characters showed a hump-shaped or monotonical increase with increasing mutation rate (Fig 5). The differences among these patterns of variance reflect the differences in the variances of phenotypic plasticity. Thus, the variance of phenotypic plasticity became rather high at low mutation rates (5× 10−5–5 × 10^−6^). The shape of the tree was the most complex at the 5 × 10^−5^ mutation rate, and became rather simple at the high mutation rate.

**Fig 6:**
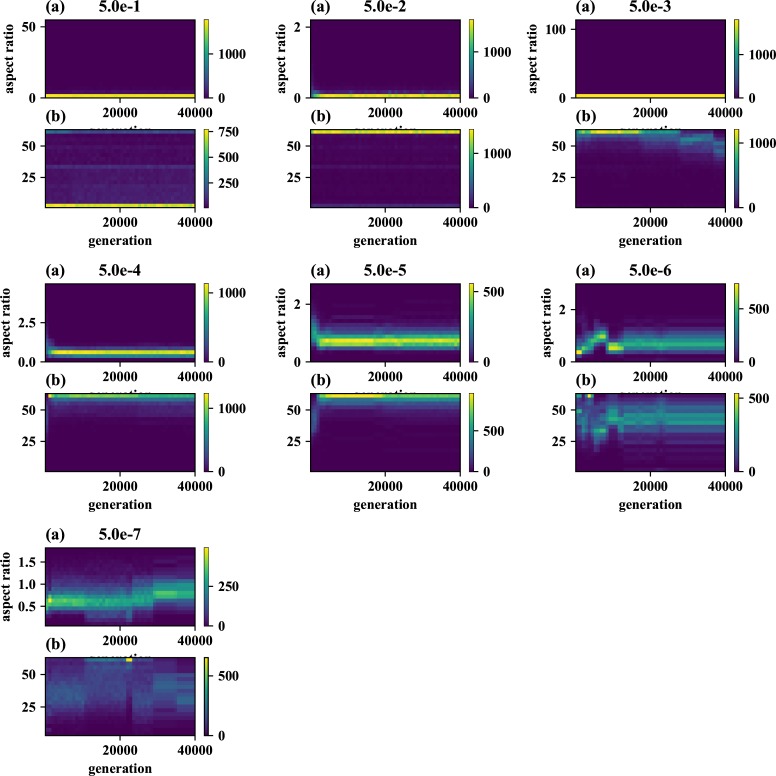
The phenotypic change through 40000 generations. (a) Horizontal axis represents the generations, and vertical axis represents the aspect ratio, which is calculated by *D*_*v*_/*D*_*h*_, where *D* is the distance from the root to the furthest branch, and small v and h denote the horizontal distance and vertical distance. (b) Horizontal axis represents the generations, and vertical axis represents the branch number. The results are sampled every 100 generations from 100 generations until 40000 generations. Mutation rates are indicated in each panel.

**Fig 7:**
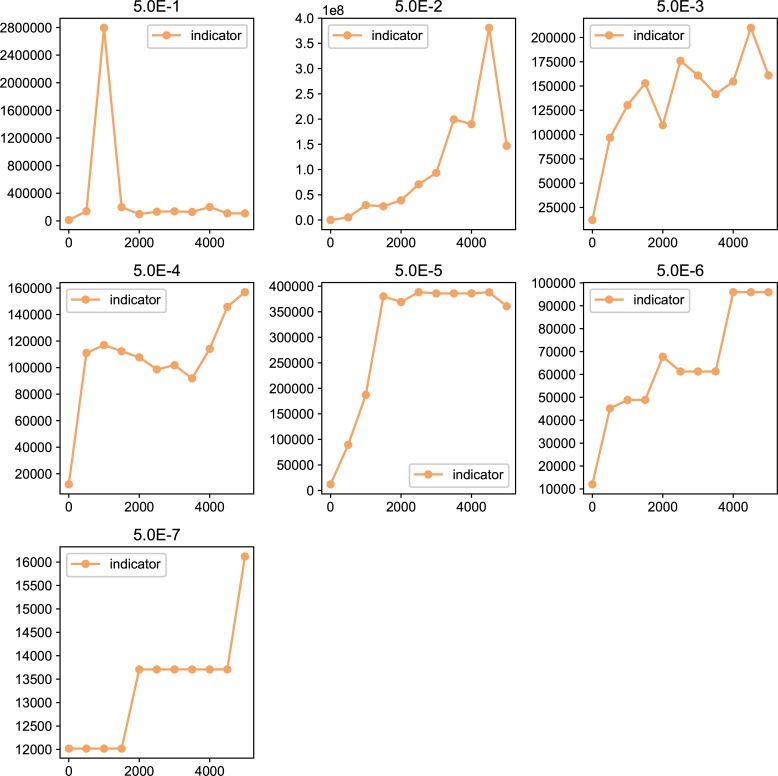
The highest fitness in each results after 5000 generations. Horizontal axis represents the generations, and vertical axis represents the fitness. Each dot in the graph represents the highest fitness in each generations. The fitness is sampled in every 100 generations.

**Fig 8:**
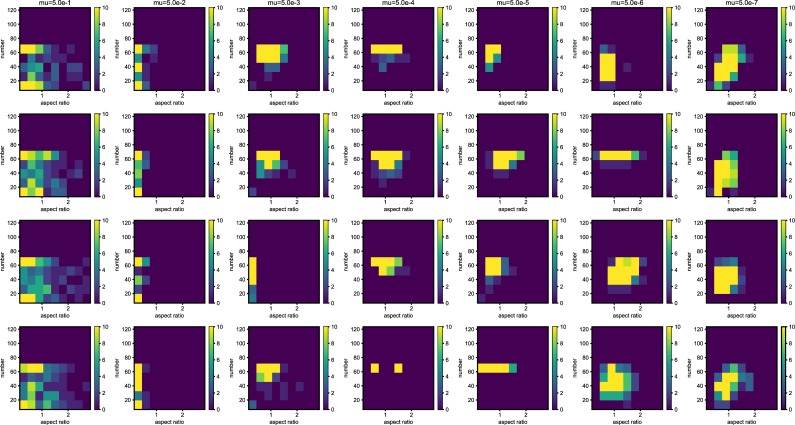
The phenotypic histogram of the whole result. Each map is one experiment result, and the horizontal axis represents aspect ratio, which is calculated by *D*_*v*_/*D*_*h*_, where *D* is the distance from the root to the most furthest branch, and small v and h denote the horizontal distance and vertical distance. The vertical axis represents the number of the branches in the individual. The six columns represent the mutation rates. The mutation rates were 5.0*e*−1, 5.0*e*−2,5.0*e*−3, 5.0e−4,5.0e−5,5.0e−6,5.0e−7 from the left. Every result contains 225 individuals after 5000 generations. The color bar of the figures are limit at 10 individuals.

**Fig 9:**
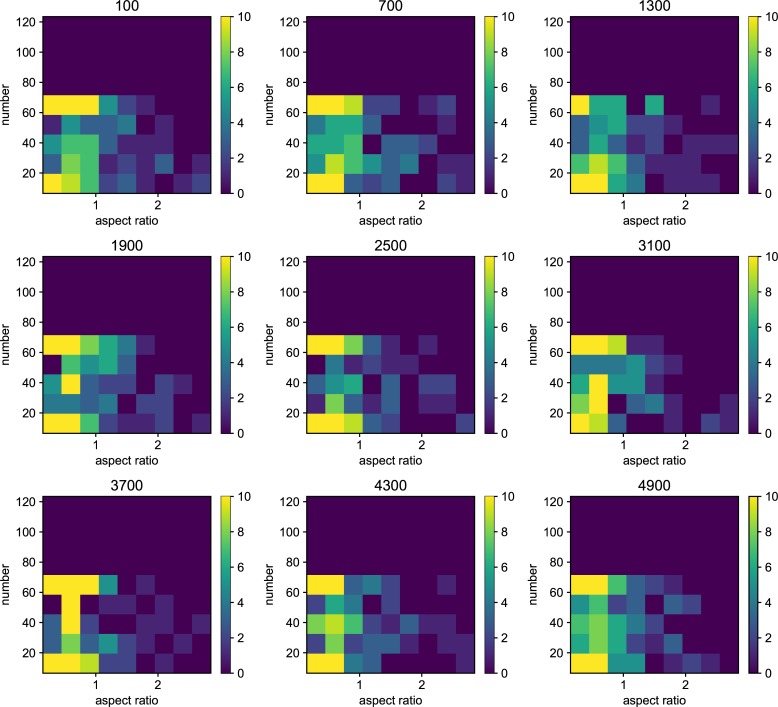
Phenotypic change along the generations when the mutation rate is 5.0*e*−1. Each map of the Figure is the midway results. The results are sampled every 500 generations from 100 generations until 4900 generations. the horizontal axis represents aspect ratio, which is calculated by *D*_*v*_/*D*_*h*_, where *D* is the distance from the root to the most furthest branch, and small v and h denote the horizontal distance and vertical distance. The vertical axis represents the number of the branches in the individual. The six columns represent the mutation rates.

**Fig 10:**
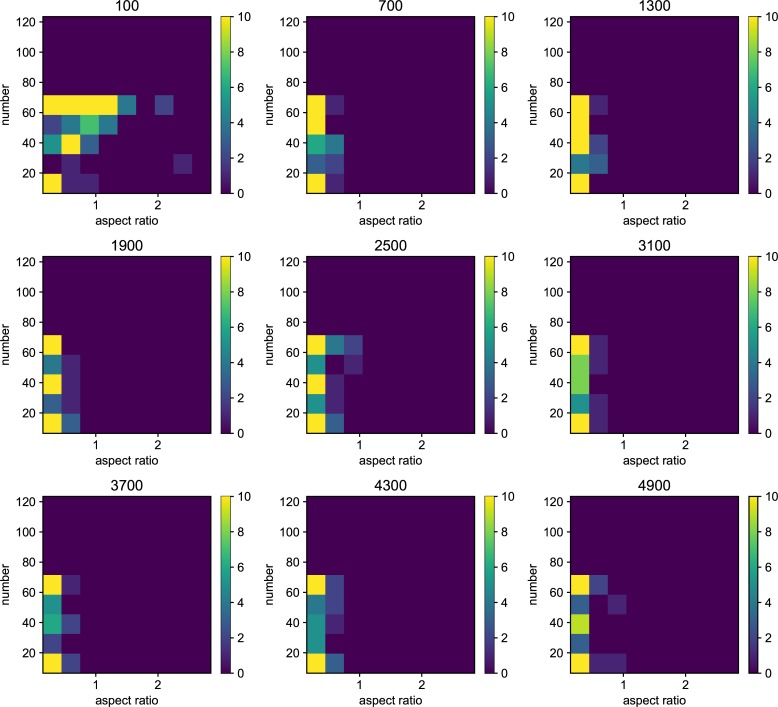
Phenotypic change along the generations when the mutation rate is 5.0*e*−2.

**Fig 11:**
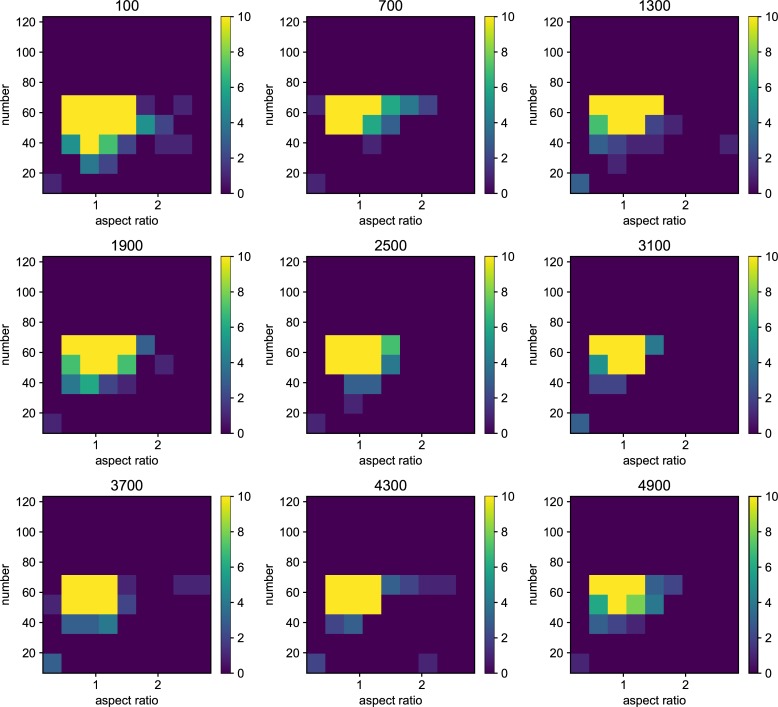
Phenotypic change along the generations when the mutation rate is 5.0*e*−3.

**Fig 12:**
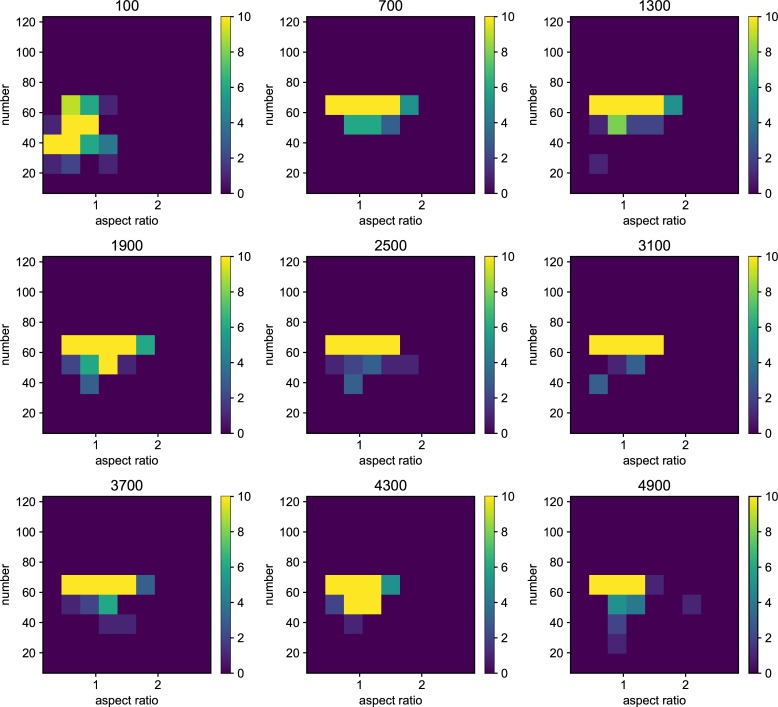
Phenotypic change along the generations when the mutation rate is 5.0*e*−4.

**Fig 13:**
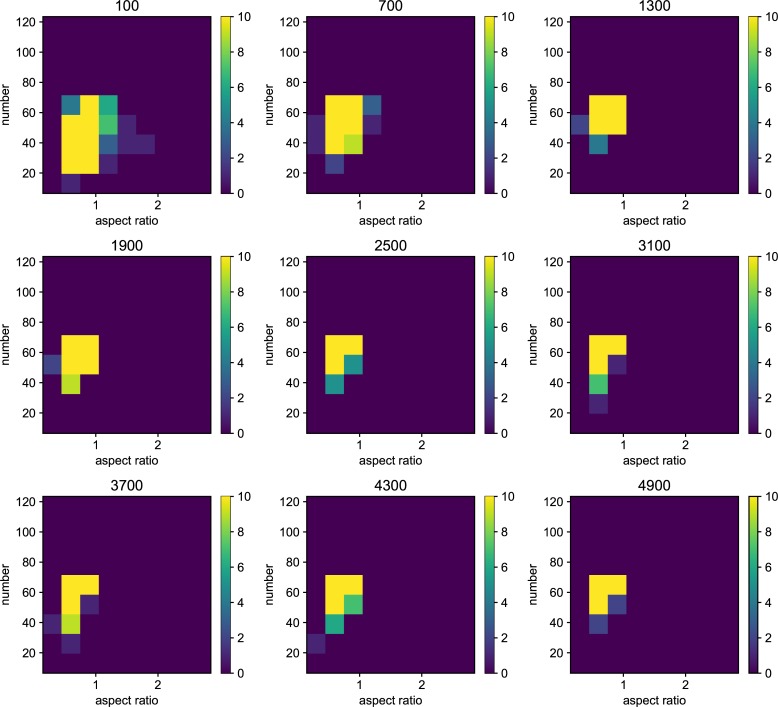
Phenotypic change along the generations when the mutation rate is 5.0*e*−5.

**Fig 14:**
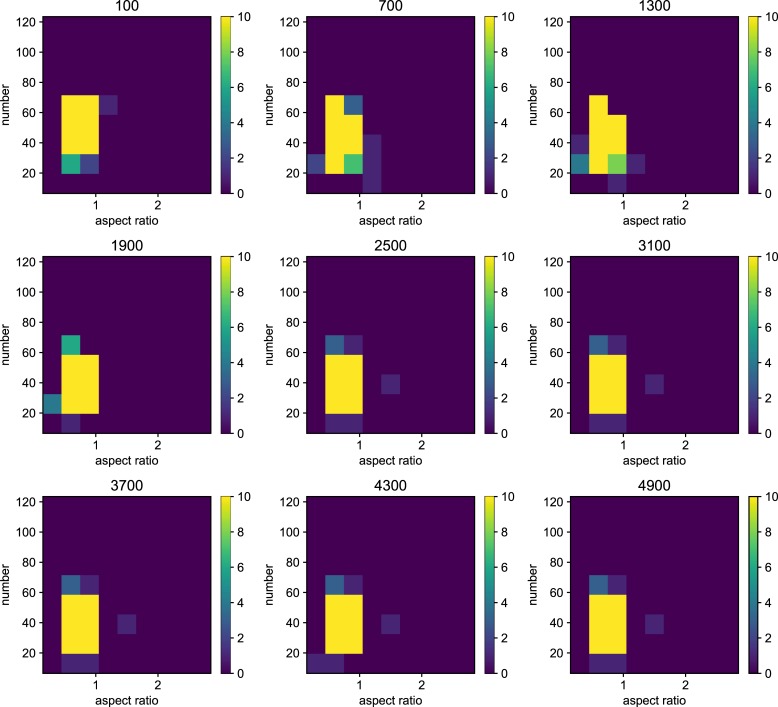
Phenotypic change along the generations when the mutation rate is 5.0*e*−6.

**Fig 15:**
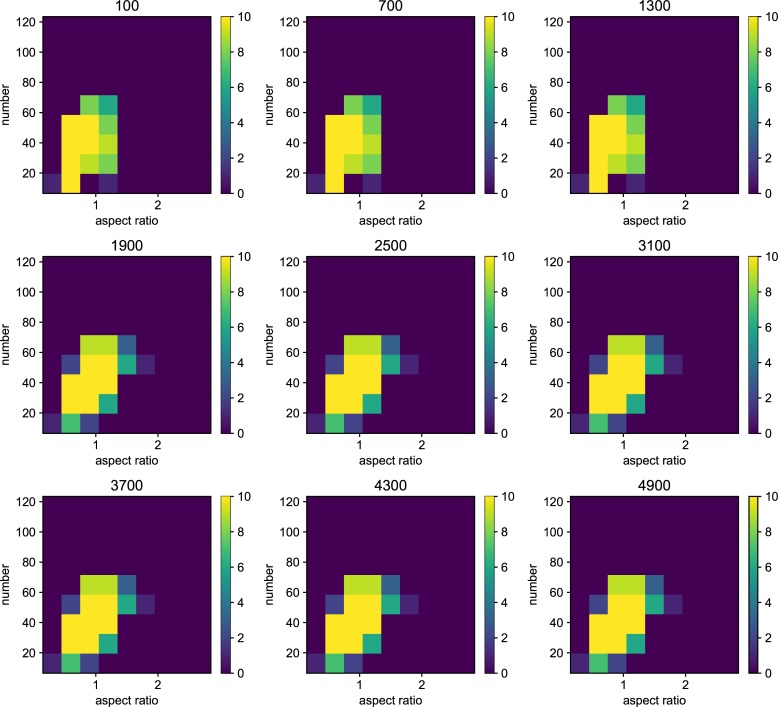
Phenotypic change along the generations when the mutation rate is 5.0*e*−7.

**Fig 16:**
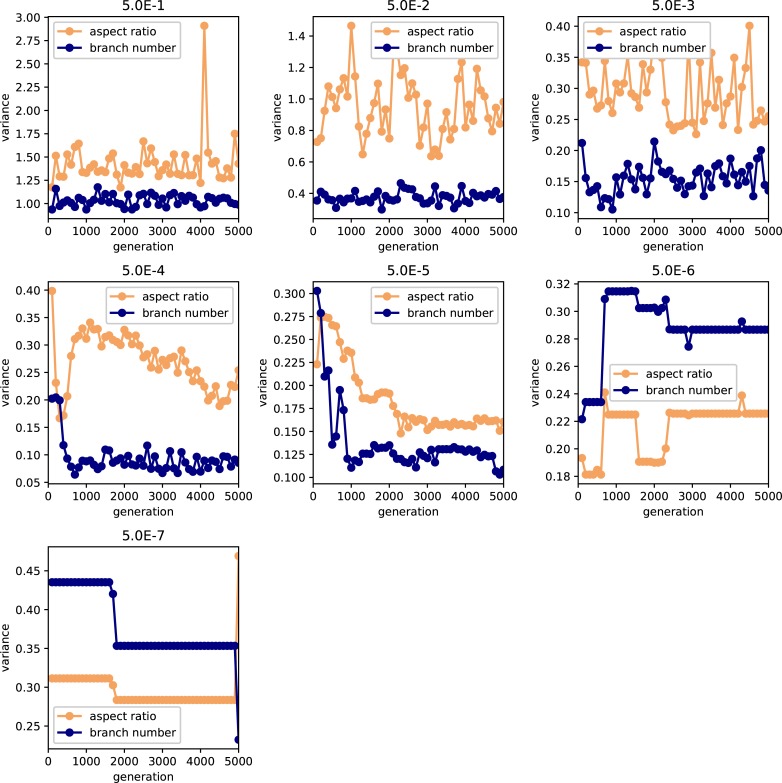
The phenotypic divergence change along the generations. The horizontal axis represents the mutation rate. The vertical axis represents the coefficient of variance of the aspect ratio and number of the branch in each individuals. The Variance of the result in aspect ratio and branch number. Orange point and line represents the aspect ratio and blue line represents the branch number. Each map represents the result in another mutation rate. The mutation rate is 5.0*e*−1, 5.0*e*−2,5.0*e*−3, 5.0e−4,5.0e−5,5.0e−6,5.0e−7, in order from the upper left.

We also examined the examined in the lower mutation rate in longer generations. We set the simulation for 225 individuals, and 40000 generations (Fig 6). The trend does not change from 5000 generations results. We show the highest fitness change over generations in Fig 7. The value reaches attenuation with the progress of generation (5× 10−2–5 × 10^−5^). In lower mutation rate, highest fitness jumps by the mutation in contingency (5× 10−6–5 × 10^−7^). Furthermore, the mutation that brings the fitness jump to the organisms does not occur in the lowest mutation rate. We examined the aspect ratio and number of the branches of the all trees generated after 5000 generations (Fig 8). We also examined two traits change until end of the simulation (Figs 9–15) in typical cases. The phenotypic tendency already determined until around 1500 generations in most of the cases. The change of the phenotypic divergence is shown in the typical cases are shown in Fig 16.

Details are the same as in Fig 9 except for mutation rate.

**Fig 17:**
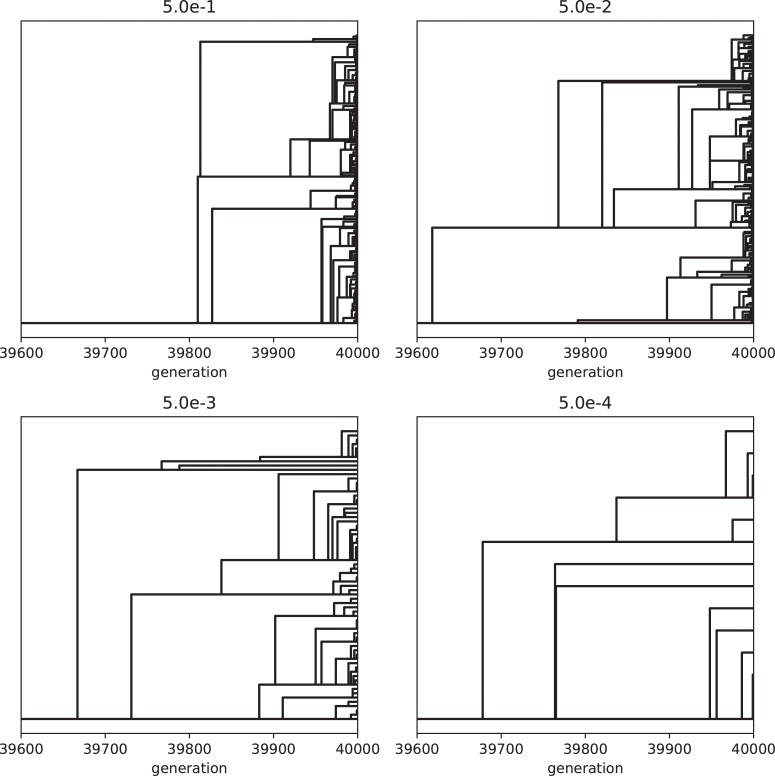
Phylogenetic relationships among lineages generated during 40000 generations. Horizontal axis represents generations. Horizontal lines indicate lineages that survived after 40000 generations. The vertical positions of the lineages do not reflect phenotypic differences among the lineages. The number on each panel indicates mutation rates. The phylogenies obtained when the mutation rates were 5× 10^−5^, 5 × 10^−6^ and 5 × 10^-7^are not shown, because only single lineage survived in these cases. Other parameters are same as the simulation in Fig 7.

Phylogenetic relationships among lineages survived after 40000 generations showed that all of these lineages diverged after or around 39600 generations (Fig 17).

## Discussion

The phenotypic patterns observed at 5000 generations are regarded as mostly equilibrium, because the trends observed through 40000 generations are mostly consistent after around 5000 generations. The phylogenetic relationships among the lineages survived at particular generations are mostly diversified from a single lineage before less than 400 generations. Although lineages are constantly replaced by newly evolved lineages, phenotypes distributions of the lineages are mostly stable through time. Thus, it is unlikely that the phenotypic patterns reflect long-term interactions among a few numbers of long lived lineages.

A high mutation rate enables the phenotype to more easily move on the surface of the adaptive landscape. Under the high mutation rate, the population consists of multiple competing clones that differ genetically from each other [33,34]. In these cases, the phenotype reaches a global peak in the adaptive landscape [13]. Therefore, it is expected that the same phenotype evolves at the end of all simulations when the mutation rate is high. In terms of the two phenotypic characters, including both effects of genetic components and plasticity (aspect ratio of branches and number of branches), the results of the present simulation are consistent with this expectation. However, in this simulation, the variances of these traits are the highest at the highest mutation rate. The variances of many of the genetically determined characters also became highest at the highest mutation rate. These findings mean that phenotypic characteristics are mostly determined by the genetic component when the mutation rate is very high. A high mutation rate (e.g., 5 × 10^−3^ and 5 × 10^−4^) causes a decrease of branch number and aspect ratio, resulting in thin and sparse individuals. Thin and sparse trees are the least susceptible to plastic change because these trees have potentially few chance to interact with other individuals than thick trees. Furthermore, high dense trees also have high plasticity, and the light amount they can get is easily increase or decrease due to light environment by chance. The number of branches is higher at the mutation rate of 5 × 10^−2^ than at lower mutation rate. Quite small and many branches were developed in several trees under the condition of this mutation rate, so that aspect ratio has not much changed relative to the lower mutation rates. Because small branches hardly interfere with the other branches, this type of tree also does not represent plastic change. In this case, the mean phenotype evolved is mostly equal among the different simulations (low *D*), but the variation of the phenotype is high due to the high mutation rate.

In contrast, a high variety of phenotypic distribution patterns (large *D*) arises at the mutation rate of 5 × 10^−5^. A low mutation rate implies that it is difficult to move a long distance on the fitness landscape [35]. The dominant phenotypes of the two traits (aspect ratio of branches and number of branches) that appeared in each simulation differ among the simulations. This pattern implies that phenotypes are trapped at particular adaptive peaks, and cannot reach a global peak of the adaptive landscape. However, in this simulation, the variances of these traits, particularly of the aspect ratio of branches, becomes rather high at the mutation rate of 5 × 10^−5^. Also, variances are low in most of the genetically determined characters at this mutation rate. Thus, the phenotypic variances arising at this level of mutation rate are mainly attributed to phenotypic plasticity. Yokozawa demonstrated that crown architecture traits are important for the pattern of species coexistence in trees [36]. Complex tree shape and high variability in tree morphology are maintained due to the plasticity.

In this paper, we assume that the traits determined by plasticity and the genome-determined traits are separated. Numbers of studies have been conducted to identify the relative contribution of genes and environment. However, developmental plasticity is partly affected by gene networks[37], therefore it is, in general, difficult to separate these two aspects[38]. Although it is difficult to classify the observed patterns in nature to either deterministic or non-deterministic, and to either genetic or environmental[39]., we tentatively used the model that can separate these processes to evaluate the importance of each factor for simplicity. Future studies incorporating more continual and complex situation of deterministic-contingent processes and plastic-geneme-determined traits.

In this simulation, phenotypic plasticity results from the interactions among branches attempting to obtain light. Such interactions among branches might yield the unique shape of the tree by chance. When the mutation rate is 5 × 10^−5^, the shape of the tree becomes complex. This suggests that strong and high frequency of interactions occur among the branches, resulting in the complex shape of the tree. Under such conditions, small spatial differences in the light environment appear by chance as a result of interaction among branches of the same and different trees. These differences might change the shape of the adaptive landscape. Thus, such plasticity is likely to promote the evolution of divergent phenotypes. Also, plasticity increases contingency, resulting in less repeatability of the evolutionary outcome among the different simulations.

When the mutation rate is very low (i.e., 5 × 10^−6^), the repeatability of the phenotypic distribution remains low. This low level of repeatability and high level of contingency are supposedly derived from the effect of phenotypic plasticity. This is because variances of the genetically determined characters are very low and near zero, but variances of the two phenotypic traits, which include variance due to plasticity, are relatively high. Thus, phenotypic plasticity again increases the contingency and yield different phenotypic distributions at the end of the different simulations.

In the present simulation, we could not separate the phenotypic variance into genetic variance and environmental variance. We estimated the effect of the plasticity on the phenotypic variance by comparing the patterns of relationship between variables to develop the tree and parameters describing the tree shape. Although the former is determined only genetically and reflects genetic variance, the latter includes both genetic and environmental variance. Therefore, to better estimate the effects of plasticity in future studies, it is necessary to develop a model that can separate genetic variance and phenotypic variance. Furthermore, the regeneration niche has been regarded as an important factor for coexisting of species [40,41]. The overlap of individual rearrangement and generation change might also affect the coexistence of species. However, the present findings still suggest that phenotypic plasticity cannot be ignored as a factor to enhance the diversity of phenotypes through the increase of contingency. Plasticity potentially plays a major role in producing phenotypic diversity with relatively low mutation rates.
